# Expression of V_1A_ and GRP receptors leads to cellular transformation and increased sensitivity to substance-P analogue-induced growth inhibition

**DOI:** 10.1038/sj.bjc.6602366

**Published:** 2005-02-01

**Authors:** A C MacKinnon, U Tufail-Hanif, C D Lucas, D Jodrell, C Haslett, T Sethi

**Affiliations:** 1 Centre for Inflammation Research, University of Edinburgh, Hugh Robson Building, George Square, Edinburgh EH8 9XD, UK; 2 Cancer Research UK, Medical Oncology Unit, Western General Hospital, Crewe Road South, Edinburgh EH4 2XU, UK

**Keywords:** SCLC, vasopressin, GRP, transformation, SP-D, SP-G

## Abstract

Small-cell lung cancer (SCLC) is a particularly aggressive cancer, which metastasises early. Despite initial sensitivity to radio- and chemo-therapy, it invariably relapses, so that the 2-year survival remains less than 5%. Neuropeptides particularly arginine vasopressin (AVP) and gastrin-releasing peptide (GRP) act as autocrine and paracrine growth factors and the expression of these and their receptors are a hallmark of the disease. Substance-P analogues including [D-Arg^1^,D-Phe^5^,D-Trp^7,9^,Leu^11^]-substance-P (SP-D) and [Arg^6^,D-Trp^7,9^,*N*^me^Phe^8^]-substance-P (6–11) (SP-G) inhibit the growth of SCLC cells by modulating neuropeptide signalling. We show that GRP and V_1A_ receptors expression leads to the development of a transformed phenotype. Addition of neuropeptide provides some protection from etoposide-induced cytotoxicity. Receptor expression also leads to an increased sensitivity to substance-P analogue-induced growth inhibition. We show that SP-D and SP-G act as biased agonists at GRP and V_1A_ receptors causing blockade of G_q_-mediated Ca^2+^ release while directing signalling to activate ERK via a pertussis toxin-sensitive pathway. This is the first description of biased agonism at V_1A_ receptors. This unique pharmacology governs the antiproliferative properties of these agents and highlights their potential therapeutic potential for the treatment of SCLC and particularly in tumours, which have developed resistance to chemotherapy.

Lung cancer is the most common fatal malignancy in the developed world. Small-cell lung cancer (SCLC), which constitutes 25% of the total, is a particularly aggressive form of lung cancer. It metastasises early and over 90% of patients have widespread metastasis at presentation precluding curative surgery. Despite initial sensitivity to radio- and chemo-therapy, SCLC almost invariably relapses, so that the 2-year survival remains less than 5% (Smyth *et al*, 1986). Small-cell lung cancer is a paradigm for neuropeptide-driven tumourigenesis. Small-cell lung cancer cells proliferate in response to a range of neuropeptide growth factors and in many cases these neuropeptides are involved in autocrine and paracrine growth loops, which fuel unrestrained proliferation (Moody *et al*, 1981; Sethi and Rozengurt, 1991; Sethi *et al*, 1992; North, 2000). This is thought to be part of a special process of oncogenic transformation called Selective Tumour gene Expression of Peptides essential for Survival (STEPS) (North, 2000). The expression of neuropeptides and their receptors in tumour cells may render them sensitive to pharmacological therapeutic intervention and may be of diagnostic importance for the early detection of lung cancer and for the selection of appropriate treatment. Much of the recent studies in SCLC have focused on the roles of the specific mitogens gastrin-releasing peptide (GRP, the mammalian homologue of bombesin) and arginine vasopressin (AVP).

Gastrin-releasing peptide receptors are frequently aberrantly expressed in human neuroendocrine tumours of the breast, prostate, lung and colon (Carroll *et al*, 1999; Jensen *et al*, 2001) where they have both mitogenic and morphogenic roles and confer a survival advantage in proliferating cancer cells. It has been suggested that the detection of these markers in the peripheral blood of patients may be useful as early markers of SCLC. Scintigraphy with the labelled peptide ligand 99mTc-bombesin/GRP has been shown to detect prostate cancer and invasion of pelvic lymph nodes and should be applicable to other endocrine tumours particularly SCLC (De Vincentis *et al*, 2004). Monoclonal antibodies have been developed against circulating bombesin and one such antibody 2A11 has been shown to inhibit the growth of SCLC *in vitro* and as xenografts in nude mice (Chaudhry *et al*, 1999); however, it has limited efficacy in human trials.

The presence of AVP and V_1A_ receptors on SCLC tumours has been extensively studied and the potential presence of an autocrine growth loop has been established. Small-cell lung cancer patients frequently display symptoms of inappropriate antidiuretic hormone secretion such as hyponatremia and urinary hyperosmolality (Johnson *et al*, 1997). Independent studies have shown the expression of V_1A_ receptors in five out of five SCLC lines and zero out of four non-small-cell lung cancer (NSCLC) lines (Ocejo-Garcia *et al*, 2001) while we showed expression of V_1A_ receptors in four out of four SCLC lines (Waters *et al*, 2003). Expression of V_1A_ receptors and vasopressin is the most useful diagnostic tool for differentiating SCLC from NSCLC and other cancers (Coulson *et al*, 2003). Therapy that targets V_1A_ receptors may be of potential benefit for SCLC. Small-cell lung cancer cells can also express the AVP gene as provasopressin, which remains attached to the cell membrane and conceivably contributes to the autocrine-driven mitogenesis (Friedmann *et al*, 1994). Antibodies recognising this cell surface antigen have been developed as a potential diagnostic and therapeutic tool that targets SCLC tumours *in vivo* (Keegan *et al*, 2002).

However, the main focus of research in this drug development strategy has been in the development of broad-spectrum neuropeptide antagonists. Synthetic analogues of substance-P for example, [Arg^6^,D-Trp^7,9^,*N*^me^Phe^8^]-substance-P (6–11) (SP-G) were initially identified as antagonists of substance-P-mediated cellular effects and were subsequently found to also antagonise the cellular effects of bombesin (Jensen *et al*, 1984). SP-G and its analogue SP-D inhibit calcium mobilisation stimulated by bombesin and vasopressin in SCLC cells (Woll and Rozengurt, 1988; Langdon *et al*, 1992; Sethi *et al*, 1992). They also inhibit mitogenesis by the same neuropeptides in both Swiss 3T3 cells and SCLC cells (Woll and Rozengurt, 1988; Sethi *et al*, 1992; Seckl *et al*, 1995) In addition to the *in vitro* growth inhibitory effects of substance-P analogues, these compounds inhibit the growth of tumours in xenograft models in nude mice (Langdon *et al*, 1992) and are more effective than specific neuropeptide receptor antagonists. SP-G has recently completed a phase I clinical trial where it shows minimal toxicity at therapeutic plasma concentrations (Clive *et al*, 2001). However, its mechanism of action is still being investigated.

Our previous work has shown that SP-G and SP-D do not act as simple competitive antagonists of GRP receptors but rather act as biased agonists, inhibiting GRP-stimulated PLC activation via G_q_ while directly stimulating JNK and ERK via G_12_ and G_i_, respectively (Tallet *et al*, 1996; Jarpe *et al*, 1998; MacKinnon *et al*, 1999, 2001) The prolonged stimulation of JNK and ERK coupled with an inhibition of intracellular Ca^2+^ is fundamental for the antiproliferative and proapoptotic effects of these agents (MacKinnon *et al*, 2001, Waters *et al*, 2003). It is not known whether these compounds interact with vasopressin receptors in the same fashion.

It was the objective of the present study to examine the effect of GRPr and V_1A_r expression, the two most common neuropeptide receptors present on SCLC, on growth and transformation of the epithelial cell line, CHO-K1. We show that expression of these receptors leads to an increase in basal and anchorage-independent growth in low serum, and an increased sensitivity to substance-P analogues. As well as directing GRP receptor signalling, substance-P analogues also act as biased agonists at V_1A_ vasopressin receptors. These combined effects will be useful for SCLC therapy and particularly in targeting well-differentiated tumours, which have developed resistance to chemotherapy.

## METHODS

### Materials

CHO-K1 cells were purchased for the European Cell Culture Collection; Dulbecco's modified Eagle's medium (DMEM), GRP, bombesin and monoclonal antibody to diphosphorylated ERK 1 and 2 (M 8159) were from Sigma (Poole, UK); ([D-Arg^1^,D-Phe^5^,D-Trp^7,9^,Leu^11^]-substance-P (SP-D) and [D-Arg^6^,D-Trp^7,9^,*N*^me^Phe^8^]-substance-P (6–11) (SP-G) were synthesised by Cancer Research UK (London, UK). The human V_1A_ receptor construct in pcDNA3.1 was a kind gift from M Thibonnier (Case Western Reserve University School of Medicine, OH, US). The human GRP receptor in pcDNA3.1 was from J Battey (Albert Einstein College of Medicine, NY, USA). [^125^I]-Arginine vasopressin (2000 Ci mmol^−1^) and [^125^I]-GRP were from Amersham International (Amersham, UK).

### Cell culture and transfection

CHO-K1 cells were maintained in DMEM supplemented with 10% (v v^−1^) foetal bovine serum (heat-inactivated at 57°C for 1 h) 50 U ml^−1^ penicillin, 50 *μ*g ml^−1^ streptomycin and 5 *μ*g ml^−1^ L-glutamine in a humidified atmosphere of 5% CO_2_:95% air at 37°C. CHO-K1 cells were transfected with full-length human GRP receptor or human V_1A_ receptor using lipofectamine plus (Invitrogen) as per the manufacturer's instructions. Stable cell cultures were maintained in the presence of 400 *μ*g ml^−1^ G418-sulphate.

### Receptor binding

Receptor binding was carried out in confluent and quiescent cultures of CHO-K1-V_1A_r and GRPr cells in a binding medium containing DMEM, 1 mg ml^−1^ bovine serum albumin and radioligand (1.0 nM GRP containing 2nCi [^125^I]-GRP or 0.6 nM AVP containing 45 nCi [^125^I]-AVP. Incubations were carried out at 37°C for 30 min in the presence of inhibitors as indicated. Nonspecific binding was defined in the presence of 1 *μ*M GRP or AVP, respectively. The reaction was stopped on ice and unbound ligand was removed by washing × 3 with ice-cold phosphate-buffered saline. After solubilisation in 0.1 M NaOH, 2% Na_2_C0_3_ containing 1% SDS, bound ligand was estimated by liquid scintillation counting. The binding parameters *K*_d_ and *B*_max_ were calculated from competition binding isotherms with unlabelled ligand (DeBlasi *et al*, 1989). The IC_50_ (concentration of drug displacing 50% specific binding) was converted to the inhibitory constant (*K*_i_), where *K*_i_=IC_50_/(1+[ligand]/*K*_d_) (Cheng and Prusoff, 1973).

### Growth assays

#### Liquid growth

Exponentially growing CHO-K1 cells were trypsinised and suspended in DMEM with 5% FCS at a density of 5 × 10^4^ cells per plate in the presence or absence of mediators in triplicate. Cells were grown for 1–9 days and cell number determined using a Coulter Counter (model Z1, Coulter).

#### MTT assay

In some assays, MTT (3-[4,5-dimethylthiazol-2yl]-2,5-diphenyl tetrazolium bromide) formazan production (Sigma) was used to measure proliferation as per the manufacturer's instructions.

#### Clonogenic assay

CHO-K1 (2x10^4^) viable cells were mixed with DMEM containing 0.3% agarose in the presence or absence of mediators and layered over a solid base of 0.5% agarose in DMEM in 35 mm plastic dishes. The cultures were incubated at 37°C for 1–10 days, and then stained with 1 mg ml^−1^ nitro-blue tetrazolium (NBT, Sigma) overnight at 37°C. Colonies from 10 separate fields were counted using a microscope with a × 4 objective. Cloning efficiency is calculated as the percentage of original number of seeded cells forming colonies of >6 cells.

#### Aggregation assay

CHO-K1 cells were suspended in DMEM in the presence of 5% FCS and seeded into low adhesion tissue culture plates on top of a layer (1 ml) of 0.5% agar. Under these conditions, the cells did not adhere. Cells were maintained in culture for 7 days briefly trypsinised to disaggregate clusters and viable cells counted.

### Determination of intracellular Ca^2+^ concentration

CHO-K1 cells expressing the GRP or V_1A_ receptor were grown to confluence on 10 cm plates and quiesced overnight in DMEM containing 0.1%. FCS. Cells were trypsinised and loaded with fura-2-tetraacetoxymethylester AME (FURA-2-AM, 1 *μ*M) in calcium-free Hank's balanced salt solution for 10 min at 37°C. The cells were pelleted and resuspended in 2 ml of Hank's balanced salt solution containing 1.8 mM CaCl_2_. Fluorescence was recorded in a fluorescence spectrophotometer (Perkin Elmer). Alternate dual wavelength excitation at 380 and 410 nm allowed ratiometric analysis of bound and unbound FURA-2AM when measured at 505 nm. [Ca^2+^] was calculated according to the equation [Ca^2+^]=*K*(*F*−*F*_min_)/(*F*_max_−*F*), where *F* is the ratio of the unknown sample, *F*_max_ is the ratio after the addition of 0.1% Triton X-100 and *F*_min_ is the ratio after Ca^2+^ chelation with 10 mM EGTA. *K* is the dissociation constant for Fura-2, which is 224 nM.

### Western blotting

Quiescent cell cultures in six-well plates were treated as described in figure legends and lysed at 4°C in 0.25 ml lysis buffer containing; 25 mM HEPES pH 7.4, 0.3 M NaCl, 1.5 mM MgCl_2_, 0.2 mM EDTA, 0.5% Triton X-100, 20 mM *β*-glycerphosphate, 0.5 mM dithiothreitol, 1 mM sodium orthovanadate and protease inhibitors (Boehringer Mannheim, Sussex, UK; prepared as per the manufacturer's instructions). Lysates were clarified by centrifugation, equilibrated for protein using BCA protein assay reagent (Perbio Science, Cheshire, UK) and denatured by boiling (5 min) in SDS–PAGE loading buffer. In all, 20 *μ*l lysate/lane was resolved on 12% SDS–PAGE gels and electroblotted onto nitrocellulose membranes. Membranes were blocked in 3% bovine serum albumin in PBS containing 0.05% Tween-20. ERK_1/2_ phosphorylation was determined using 1 : 1000 dilution of the primary antibody followed by the appropriate HRP-labelled goat IgG (DAKO, UK) diluted 1 : 5000. Bands were visualized using enhanced chemiluminescence (ECL plus, Amersham) and quantified using a phosphor imager (Storm).

## RESULTS

### Receptor binding

**Figure 1:**
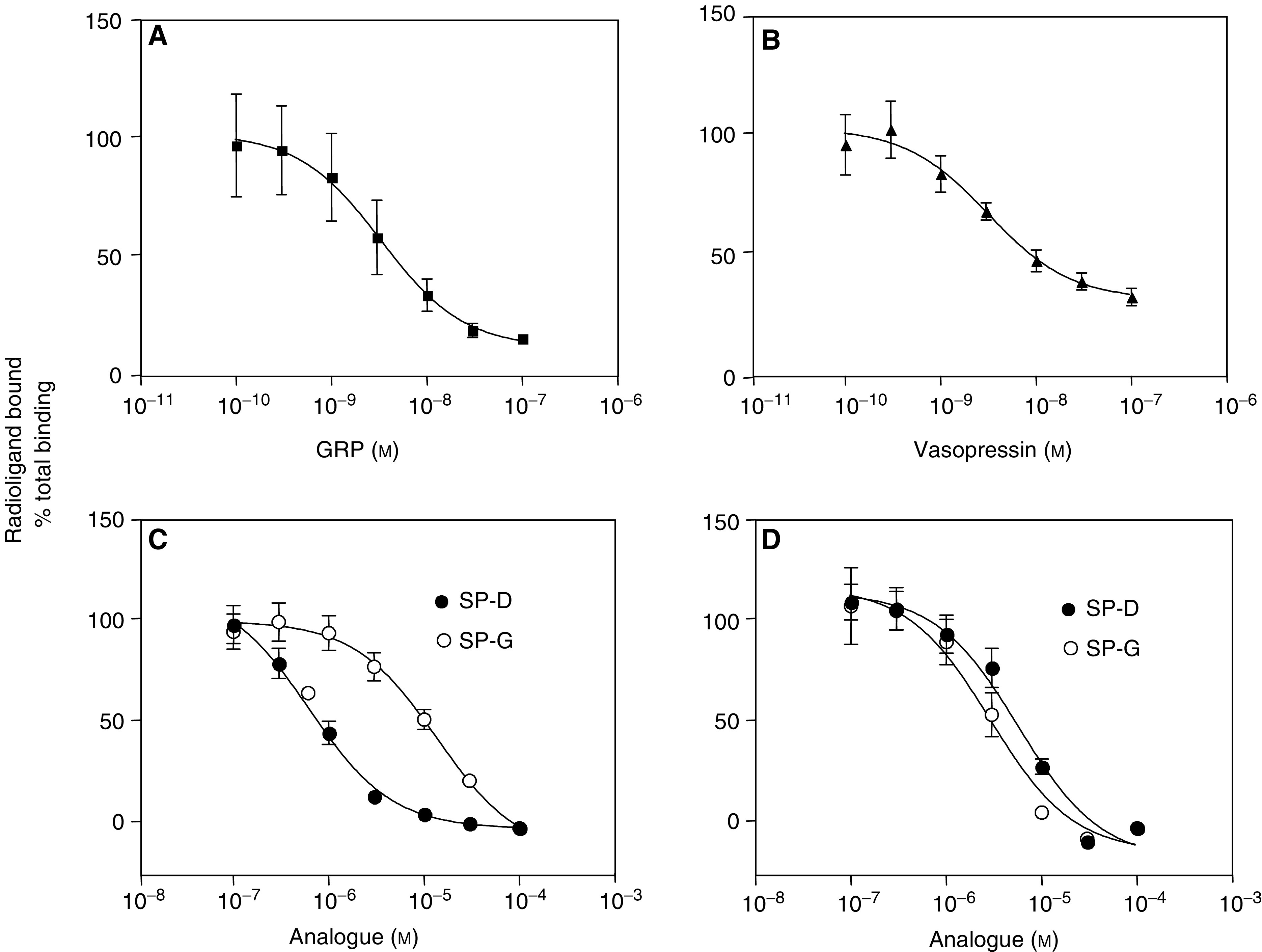
Receptor binding. Gastrin-releasing peptide (**A**, **C**) or V_1A_ (**B**, **D**) receptor expressing CHO cells were incubated with [^125I^]-GRP or [^125^I]-AVP for 30 min at 37°C as described in Methods in the presence of various concentrations of unlabelled GRP (**A**), unlabelled AVP (**B**), SP-D or SP-G (**C**, **D**). Results are expressed as % total binding and represent the mean±s.e.m. of four experiments performed in triplicate.

To assess receptor expression, stable cultures of CHO-K1-GRP and CHO-K1-V_1A_ cells were incubated with the appropriate radioligand, and receptor number and affinity were measured from competition binding curves with unlabelled ligand. Figure 1 shows that GRP receptors were expressed with *K*_d_=2.55±0.84 nM and *B*_max_ of 1151±326 sites/cell (*n*=4). V_1A_ receptors were expressed at a slightly lower level with a *B*_max_=450±80 sites/cell and *K*_d_=2.98±0.71 nM (*n*=4, Figure 1B). The substance-P analogues SP-G and SP-D inhibited [^125^I]-GRP binding with affinities in the micromolar range (SP-G *K*_i_=19.4±6.3 *μ*M (*n*=4), SP-D *K*_i_=0.64±0.05 *μ*M (*n*=4, Figure 1)). Both analogues also inhibited V_1A_ receptor binding ((SP-G *K*_i_=3.50±0.82 *μ*M (*n*=4), SP-D *K*_i_=8.58±1.47 *μ*M (*n*=4)). This data show that SP-G is relatively (six-fold) more selective for the V_1A_ receptor whereas SP-D is GRP receptor selective (13-fold).

### Effects of V_1A_ and GRP receptor expression on cell growth in liquid culture and semisolid media

**Figure 2:**
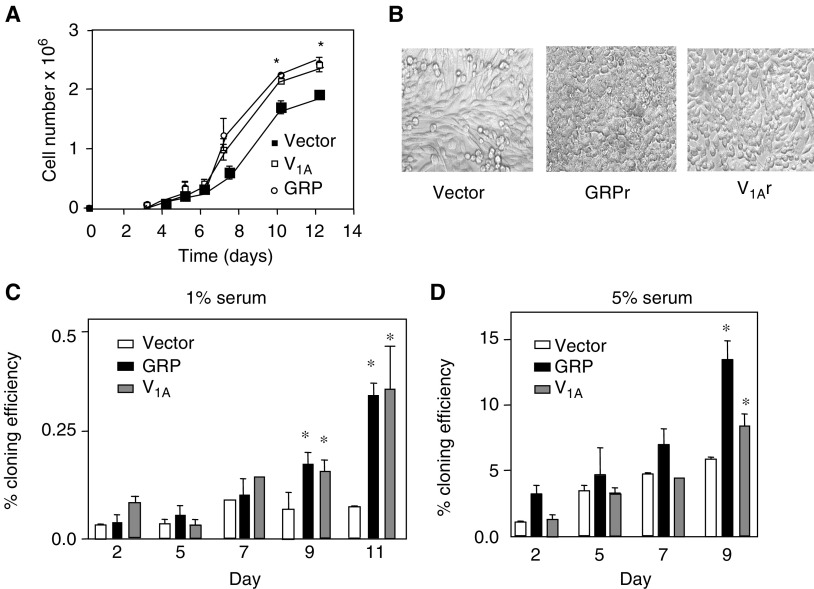
Effect of neuropeptide receptor expression on cell growth *liquid growth* (**A**) CHO-K1 cells expressing vector, the GRP or V_1A_ receptor were plated at a density of 1e^4^ cells/100 mm tissue culture dish and incubated at 37°C. Cells were harvested at various times and counted. Results represent the mean+s.e.m. of three experiments performed in duplicate (^*^significantly different from vector control cells *P*<0.05, ANOVA). (**B**) Representative images showing morphological features of transfected cells are shown. The rounded cobblestone features and higher cell density are evident in neuropeptide receptor expressing cells compared to the elongated contact inhibited monolayer exhibited by vector transfected controls. *Clonal growth* vector (open bars), GRP receptor (black bars) and V_1A_ receptor (grey bars) transfected cells were plated at 1e^4^ cells well^−1^ in 0.3% agar in DMEM containing 1% (**C**) or 5% (**D**) FCS. At various time points, cells were stained with MTT and colonies counted at × 10 magnification. Results represent the mean±s.e.m. of three experiments performed in duplicate (^*^significantly different from vector control cells *P*<0.05, ANOVA).

**Figure 3:**
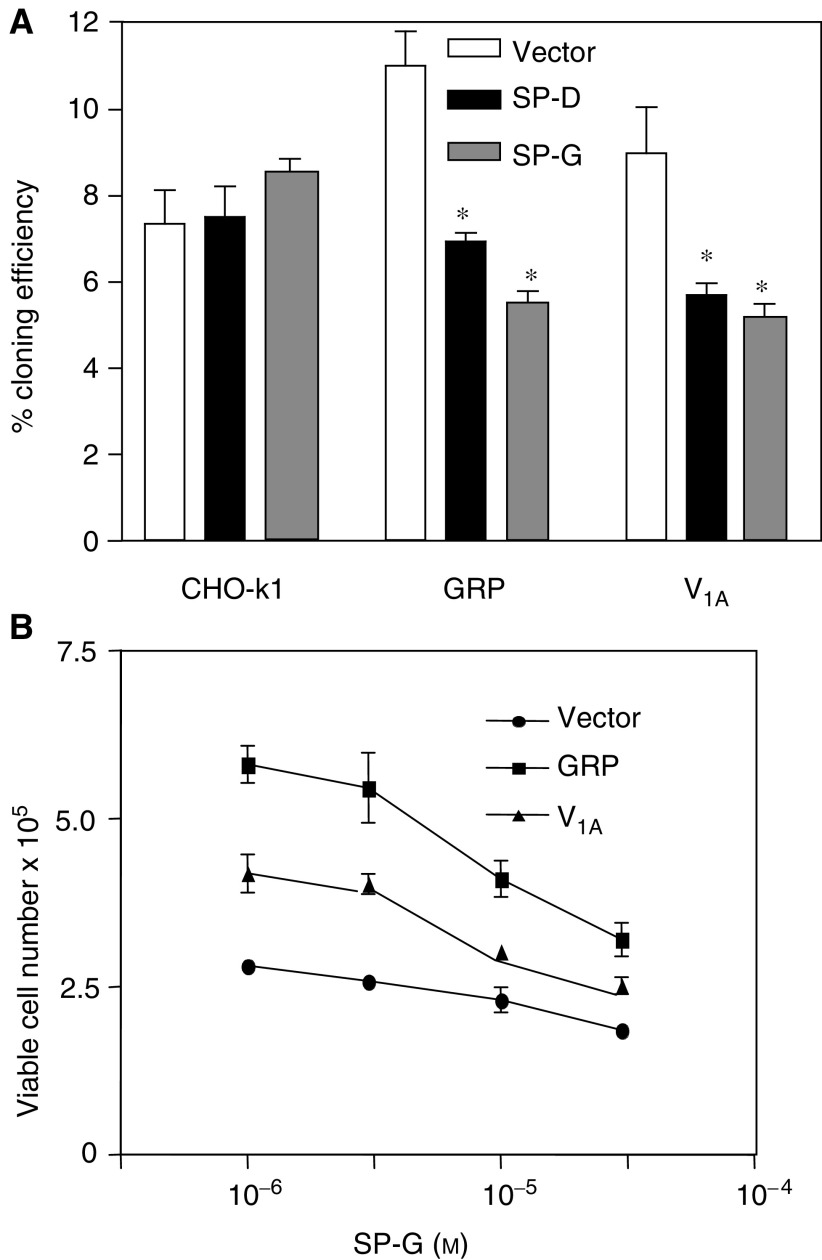
Effect of SP-D and SP-G on clonal growth. (**A**) Cells were grown in 0.3% agar with 1% FCS for 8 days in the presence or absence (open bar) of 30 *μ*M SP-D (black bar) or SP-G (grey bar). Results represent the mean±s.e.m. of three experiments performed in duplicate (^*^significantly different from wild-type controls *P*<0.05, ANOVA). *Aggregation assay*. (**B**) Wild-type, GRPR-transfected and V_1A_R-transfected CHO-K1 cells were plated into low adhesion tissue culture plates on top of a layer of 0.5% agar in DMEM containing 5% FCS at a density of 5 × 10^4^ ml^−1^ in the presence of varying concentrations of SP-G. Cells were maintained in culture for 7 days, briefly trypsinised to dissagregate clusters and viable cells counted by propidium iodide exclusion.

Figure 2A shows that in liquid culture, in the presence of 5% FCS, the GRP and the V_1A_ expressing cells proliferated at a similar rate to untransfected CHOs, but on day 6 after seeding, the GRP cells had reached a density of 4.5 × 10^6^ cells per 100 mm dish. The cells did not appear to be contact inhibited and exhibited a rounded morphology. Cells over grew one another and significant numbers of cells were observed to be detached and growing in clusters (Figure 2B). Trypan blue exclusion revealed that the cells were >95% viable. A similar, though less pronounced, effect was observed with the V_1A_-transfected cells, which reached a density of 3.1 × 10^6^ cells per 100 mm dish. The parental cell line exhibited normal contact inhibition and had flat fibroblast morphology and did not show significant detachment. The end point density of these cells was 2.1 × 10^6^ cells per 100 mm dish. These observations suggested that transfection of GRP and V_1A_ receptors caused CHO-K1 cells to lose contact inhibition and adopt a more transformed phenotype. These findings led us to examine their growth in semisolid media, which is widely regarded as being a hallmark of the transformed phenotype. Cells were trypsinised and suspended in 0.3% agarose in DMEM (1 × 10^4^cells ml^−1^) layered over a 0.5% layer of agarose in DMEM. Figure 2C shows that at day 9 after seeding, clusters of cells (>6 cells) were visible with MTT staining. The cloning efficiency was significantly higher in the GRP-expressing cells (14.1±1.5%, *P*<0.05, ANOVA) and the V_1A_ receptor-expressing cells (8.5±1.0%, *P*<0.05, ANOVA) than the vector control cells in the presence of 5% FCS (5.5±0.02%). In a separate series of experiments, the effects of SP-D and SP-G were examined (Figure 3A). At 30 *μ*M, both analogues significantly reduced colony formation in the GRP and V_1A_ receptor expressing cells but had no effect on the cloning efficiency of vector control CHO-K1 cells. The effect of receptor expression was also examined in an aggregation assay where cells are prevented from adhering and form aggregates in suspension. After 6 days, cells are briefly trypsinised and viable cells counted. These experiments showed that expression of the GRPr and V_1A_r increased anchorage-independent growth compared to wild-type cells or vector control cells. Similarly SP-G and SP-D were able to inhibit cell growth in receptor expressing cells (Figure 3B). Together, these data suggest that expression of the GRP and V_1A_ receptors induce a more transformed phenotype in CHO cells and induce sensitivity to substance-P analogue-induced growth inhibition.

### Chemosensitivity

**Figure 4:**
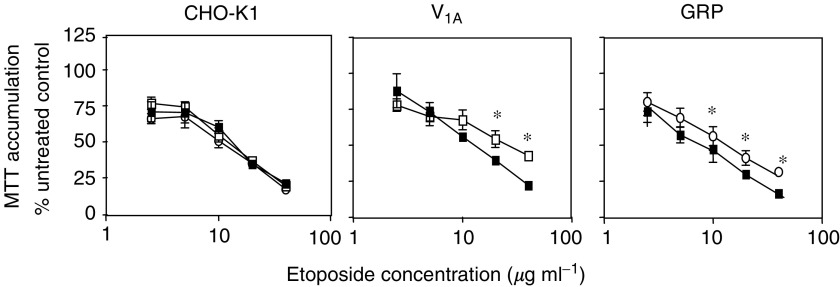
Effect of neuropeptide on chemosensitivity. Wild-type CHO-K1 cells (left) and cells expressing the V_1A_ (middle) or GRP (right) receptor were plated at a density of 1e^4^ cells per well of a 96-well tissue culture plate in DMEM with 10% FCS and incubated overnight 37°C. Cells were then incubated in serum-free media containing etoposide as indicated and in the absence (filled squares) or presence of either 50 nM AVP (open squares) or 50 nM bombesin (open circles) or both neuropeptides (wild-type cells) for 48 h at 37°C. Cell viability was assessed by MTT staining. Results are expressed as % viability in the absence of neuropeptide and are mean+s.e.m. of four independent experiments (^*^significantly different from untreated etoposide control, *P*<0.05 ANOVA).

The response to etoposide in control and receptor-transfected cells was measured by MTT accumulation. Figure 4 shows that after 48 h in culture in the absence of serum, etoposide produced a dose-dependent inhibition of proliferation in all cell types (IC_50_=12.4±3.1, 8.1±2.3 and 14.2±4.0 *μ*g ml^−1^ in CHO-WT, CHO-GRPr and CHO-V_1A_r cells, respectively). Incubation with 50 nM of either bombesin or AVP added to CHO-GRPr or CHO-V_1A_r produced a small but significant protection from etoposide, which was not observed when both neuropeptides were added to wild-type cells (IC_50_=13.0 and 26.9 *μ*g ml^−1^ in control and AVP-treated V_1A_r-expressing cells, respectively; and 6.30 and 12.7 *μ*g ml^−1^ in bombesin-treated GRPr-expressing cells, respectively). At 40 *μ*g ml^−1^ etoposide, vasopressin-treated V_1A_r-expressing cells accumulated 92% more MTT than control cells (*P*<0.01). In GRPr-expressing cells, bombesin treatment caused an increase of 52% MTT accumulation compared to untreated cells (*P*<0.01). These results suggest that neuropeptide receptor stimulation may contribute to an increase in chemoresistance.

### Intracellular [Ca^2+^]_i_

**Figure 5:**
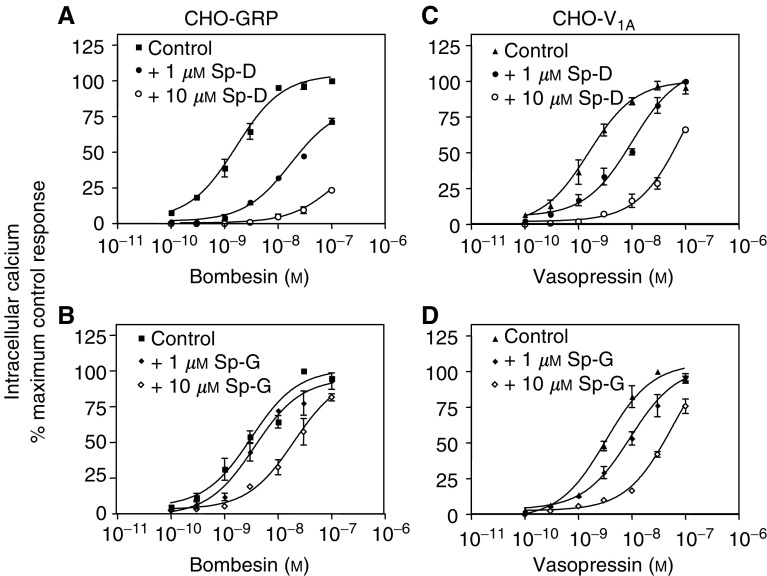
Intracellular calcium release. Quiescent GRP (**A**, **B**) or V_1A_ (**C**, **D**) receptor expressing CHO cells were incubated with FURA-2AM for 15 min at 37°C and ratiometric fluorescence monitored at 37°C. Concentration–response curves to bombesin were carried out in control cells and in cells pretreated for 2 min with 1 or 10 *μ*M SP-D (**A**) or SP-G (**B**). Concentration–response curves to vasopressin were carried out in control cells and in cells treated with 1 or 10 *μ*M SP-D (**C**) or SP-G (**D**). Results are expressed as % maximum control response to neuropeptide and represent the mean±s.e.m. of four experiments.

The mobilization of calcium from intracellular stores is one of the earliest events triggered by neuropeptide receptor activation of G_*α*q_ leading to PLC activation and subsequent generation of IP_3_. In untransfected CHO-K1 cells, neither GRP nor AVP produced a significant change in [Ca^2+^]_i_ (data not shown). In the CHO-GRP cells, GRP produced a concentration-dependent increase in [Ca^2+^]_i_ (EC_50_=2.00±0.4 nM, *n*=5, Figure 5A). SP-D and SP-G produced no change in [Ca^2+^]_i_ on their own but inhibited GRP-induced [Ca^2+^]_i_ elevation. Figure 5A and B show that SP-D and SP-G inhibited GRP-induced [Ca^2+^]_i_ with resultant pA_2_ values of 7.21 and 5.72 for SP-D and SP-G respectively. In V_1A_-expressing cells, AVP increased calcium mobilisation with an EC_50_=3.1±0.60 nM (*n*=4, Figure 5C and D). As in the GRP-expressing cells, SP-D and SP-G did not mobilise intracellular calcium directly but inhibited the response to AVP (Figure 5C and D). SP-G was more efficacious in inhibiting the vasopressin response than the GRP response. PA_2_ values for AVP antagonism were 7.23 and 6.53 for SP-D and SP-G, respectively. Together, these data suggest that when stably transfected into CHO cells the GRP and the V_1A_ receptor effectively couple to G_*α*q_ to increase intracellular calcium and that the substance-P analogues are effective antagonists of this response.

### ERK activation

**Figure 6:**
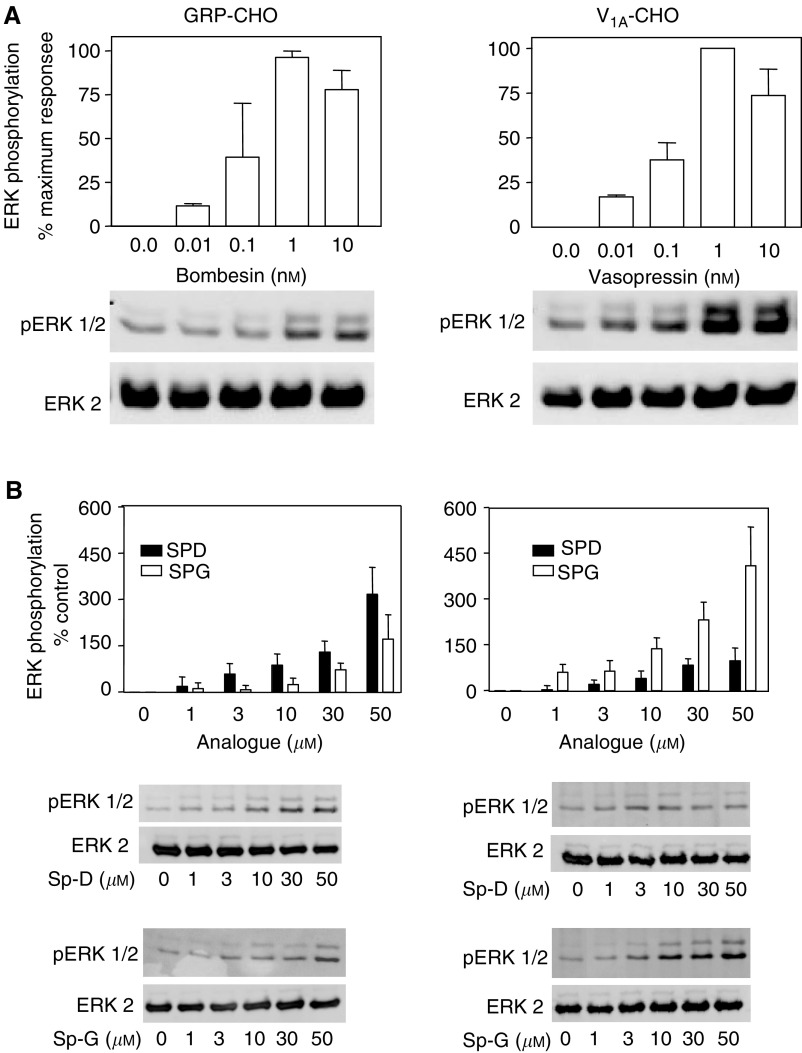
(**A**) ERK phosphorylation by neuropeptides. Quiescent GRP (left) or V_1A_ (right) receptor expressing CHO cells were stimulated for 5 min with neuropeptide at the indicated concentrations. Aliquots of cell lysate were resolved by SDS–PAGE and Western blots probed with monoclonal anti-pERK1/2 antibody (upper panel) or polyclonal anti-ERK2 antibody (lower panel). Representative Western blots are shown. Bar graphs represent the mean optical density (mean±s.e.m.) of four separate experiments. (**B**) ERK phosphorylation by substance-P analogues. Quiescent GRP (left) or V_1A_ (right) receptor expressing CHO cells were stimulated for 5 min with SP-D (filled bars) or SP-G (open bars) at the indicated concentrations. Aliquots of cell lysate were resolved by SDS–PAGE and Western blots probed with monoclonal anti-pERK1/2 antibody (upper panel) or polyclonal anti-ERK2 antibody (lower panel). Representative Western blots are shown. Bar graphs represent the mean optical density (mean±s.e.m.) of four separate experiments.

AVP and bombesin have been shown to activate extracellular regulated protein kinases (ERK 1 and 2) in a variety of cell types expressing the receptors. Much of these data suggest a G_q_- and PKC-dependent mechanism although these observations may be cell type-dependent (Della Rocca *et al*, 1999; Sinnett-Smith *et al*, 2000). Previous studies have shown that substance-P analogues reversibly inhibit ERK activation by neuropeptides in Swiss 3T3 cells; however, our studies suggest that in rat-1 fibroblasts and in human small-cell lung carcinoma cells (SCLC), in the absence of bombesin, substance-P analogues can activate ERK and c-jun kinase (JNK) in a GRP receptor-dependent manner (MacKinnon *et al*, 2001). In this study, ERK activation was measured in CHO cells expressing GRP or V_1A_ receptors in response to the appropriate neuropeptide or substance-P analogue. Activation was estimated in immunoblots from stimulated lysates using a phosphorylation state-specific antibody. Lysates from quiesced cells stimulated for 10 min with either neuropeptide or SP-D or SP-G were separated on 12% gels and blotted for pERK2 or total ERK1/2. Figure 6A shows that bombesin stimulated ERK phosphorylation in GRP-expressing CHO cells. Quantification of five separate experiments showed that bombesin produced a maximal 3.5-fold increase in ERK phosphorylation at 1 nM with an EC_50_ of 0.56 nM. In V_1A_-expressing cells, AVP produced an increase in ERK phosphorylation of 4.2-fold with an EC_50_ of 0.72 nM, which correlated with its affinity for V_1A_ receptors measured in binding studies and for the stimulation of intracellular calcium. Neither AVP nor GRP produced any stimulation of ERK phosphorylation in untransfected CHO cells (data not shown).

SP-D and SP-G stimulated ERK activation in GRP receptor expressing CHO cells (Figure 6B). Activation of ERK was evident at 3 *μ*M for SP-D, but the maximal stimulation was less than that observed with bombesin (2.8-fold for SP-D and 2.1-fold for SP-G at 50 *μ*M). The substance-P analogues also stimulated ERK in V_1A_-expressing cells. However, in these cells SP-G was more efficacious than SP-D giving a 4.3-fold stimulation at 50 *μ*M compared to a 2-fold stimulation by the same concentration of SP-D. The observed increase in ERK phosphorylation was not due to a change in total ERK expression, as ERK1/2 immunoreactivity did not change with drug treatment. These data show that at concentrations, which block neuropeptide-stimulated calcium release, substance-P analogues are agonists for ERK activation in GRP and V_1A_ expressing CHO cells.

### Mechanisms of ERK phosphorylation

**Figure 7:**
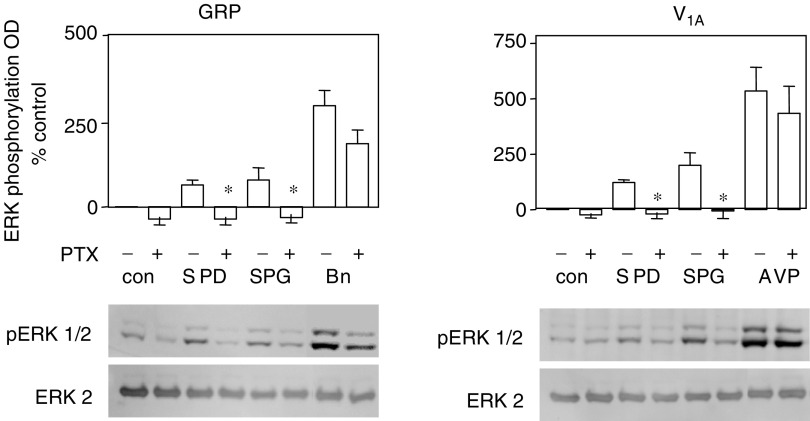
Effect of pertussis toxin (PTX) on ERK phosphorylation. Confluent cultures of GRP (left) or V_1A_ (right) receptor expressing CHO cells were quiesced overnight in the presence or absence of 100 ng ml^−1^ pertussis toxin. Cells were stimulated for 5 min with 30 *μ*M SP-D or SP-G or 1 nM neuropeptide as indicated. Aliquots of cell lysate were resolved by SDS–PAGE and Western blots probed with monoclonal anti-pERK1/2 antibody (upper panel) or polyclonal anti-ERK2 antibody (lower panel). Representative Western blots are shown. Bar graphs represent the mean optical density (mean±s.e.m.) of four separate experiments (^*^significantly different from untreated control, *P*<0.05 ANOVA).

The mechanisms of GRP- and V_1A_receptor-mediated ERK activation are not fully understood, although it has been shown in some cell types that activation is mediated by G_q_-induced PKC activation and subsequent activation of raf. Other GPCRs have been shown to activate ERK in a G_i_ and ras-dependent mechanism, which may or may not involve activation of PI3 K (Della Rocca *et al*, 1999). To examine the possible recruitment of G_i_, CHO cells were incubated overnight in quiescent media containing 100 ng ml^−1^ pertussis toxin. Figure 7 shows that in GRP receptor expressing cells, PTX produced a small but nonsignificant inhibition of bombesin-stimulated ERK activation whereas the response to 10 *μ*M SP-D was completely abolished (*P*<0.01, ANOVA). In V_1A_-expressing cells, the response to AVP was not inhibited by PTX pretreatment but the response to SP-G (20 *μ*M) was completely inhibited (*P*<0.01, ANOVA). In both cell types, ERK activation by 1 *μ*M LPA was completely inhibited by PTX treatment (data not shown). This differential sensitivity to pertussis toxin indicates that dual G_i_/G_q_-coupled mechanisms activate the ERK 1/2 cascade via GRP and V_1A_ receptors expressed in CHO cells.

## DISCUSSION

This study utilised a model CHO cell system for neuropeptide receptor expression as these cells have an acceptable null background for these receptors. The results from this work shows that (1) GRP and V_1A_ receptor expression leads to the development of a transformed phenotype in CHO-K1 cells. (2) Receptor expressing cells showed some increased resistance to the chemotherapeutic agent etoposide and increased sensitivity to the substance-P analogues, SP-D and SP-G. (3) SP-D and SP-G act as biased agonists at GRP and V_1A_ receptors. This is the first demonstration of biased agonism at vasopressin receptors. (4) This pharmacological activity is crucial for the antiproliferative effect of these agents, which may be of particular benefit in more differentiated cancers that have developed resistance to chemotherapy.

Introduction of the neuropeptide receptors into CHO cells lead to an increase in proliferation and an increase in the ability of cells to grow as colonies in semisolid medium. The cells also showed a greater propensity to grow as nonadherent clusters. Colony growth was also evident in low serum suggesting that expression of these receptors allows for anchorage- and serum-independent growth consistent with transformation. Moreover, it suggests that these receptors show some constitutive activity, as wild-type receptor expression alone was sufficient to increase transformation in the absence of exogenously added neuropeptide. The ability to observe constitutive GPCR activity is not only determined by the intrinsic molecular properties of the GPCR studied but also by the specific cellular background. Apparent constitutive activity of a wild-type GPCR may vary substantially in various systems. Differences in GPCR expression level, GPCR desensitisation, G-protein complement and GPCR/G-protein stochiometry can all contribute to such differences (Seifert and Wenzel-Seifert, 2002) This could explain why GRP receptors expressed in NCM460 cells, a nonmalignant human colon epithelial cell line, are constitutively active (Ferris *et al*, 1997) but are not in other cell types (Benya *et al*, 1994). Our findings are unlikely to be due to an overexpression artefact as the receptors in this study were expressed at levels not greater than those measured from a variety of other cancers (100–50 000 sites/cell, Jensen *et al*, 2001) So at these moderate levels, wild-type receptor was sufficient to activate downstream signals that promote cell survival under anchorage-independent conditions. The cells were, however, still able to respond to agonist, which resulted in a small but significant increase in resistance to the cytotoxic effects of etoposide. It is unclear how significant this change in sensitivity relates to the development of chemoresistance *in vivo*, but highlights an important area for future investigation.

Our previous work shows that sensitivity to AVP and GRP increases during the progression to chemoresistance in a set of SCLC cell lines (Waters *et al*, 2003). This would be analogous to the development of autocrine and paracrine growth loops in tumour development *in vivo*, with tumour cells becoming more dependent on neuropeptide growth factors for survival and subsequently developing chemoresistance. The mechanisms governing the survival advantage gained in neuropeptide receptor expressing cells are poorly understood. During colon cancer progression, increased expression of GRP receptors leads to tumour cell differentiation, increased motility and adhesion to extracellular matrix via an increased activation of focal adhesion kinase (FAK) (Glover *et al*, 2004). Previous reports have shown that GRP receptors can activate insulin-like growth factor-1 receptors (IGF-IR) leading to Akt activation in prostate cancer cells (Sumitomo *et al*, 2001), a mechanism which may underlie bombesin-mediated cell survival. In another study, human chorionic gonadotrophin (hCG) increased ovarian cell survival by up-regulating IGF-1 (Kuroda *et al*, 1998). Conversely, specific bombesin antagonists have been shown to inhibit growth and decrease EGF receptor expression in SCLC cells (Koppan *et al*, 1998) and breast cancer cells (Bajo *et al*, 2002). Bombesin antagonists in combination with GHRH antagonists have also been shown to down-regulate IGF-1, IGF-II, IGF receptors, GRP and EGF receptors in SCLC xenografts (Kanashiro *et al*, 2003). There is therefore potential for neuropeptides to regulate growth factor signalling pathways that control survival.

The present study shows that expression of GRP and V_1A_ receptors in CHO cells also leads to an increased sensitivity to substance-P analogues. This was demonstrated by an inhibition of clonal growth by substance-P analogues only in receptor-transfected cells. We previously showed that substance-P analogues are more effective at inhibiting growth of chemoresistant tumour cells expressing high GRP receptor levels and can sensitise cells to etoposide-mediated cell death (Waters *et al*, 2003). Moreover, diverse tumour types expressing GRP receptors have a greater sensitivity to SP-D (Waters *et al*, 2003). Together, these data suggest that the increase in neuropeptide receptor expression that occurs as cells become more differentiated may contribute to the development of chemoresistance and may be exploited as a target for drug therapy. The potential role of neuropeptide receptors in the development of chemoresistance is an interesting finding that requires further study.

The mechanism of action of substance-P analogues was studied in receptor-transfected cells. Upon neuropeptide receptor transfection, the cells responded to the appropriate neuropeptide as expected for these predominately G_q_-coupled receptors. The substance-P analogues behaved as antagonists for Ca^2+^ release with potencies that reflected their affinities in receptor binding experiments, with SP-G showing some selectivity for V_1A_ receptors. This may be useful in tailoring therapy to a specific tumour phenotype in as much as SP-G may be more beneficial in V_1A_ receptor expressing tumours while SP-D may be better for tumours, which express both GRP and V_1A_ receptors. Both neuropeptides and the substance-P analogues activated ERK in CHO cells expressing the GRP receptor and the V_1A_ receptor. No ERK activation was observed in untransfected CHO cells suggesting that the activation is receptor mediated. These observations suggest that substance-P analogues act as dual efficacy receptor agonists directing receptor signalling via the GRP and the V_1A_ receptor towards ERK activation while blocking liberation of intracellular calcium. Moreover, the substance-P analogues activate ERK via G_i_ as the response is pertussis toxin sensitive and more prolonged than that observed with neuropeptide (data not shown). These data support a multitrack signalling complex leading from neuropeptide receptor to ERK activation; where one track is mediated by G_q_ and most likely involves PKC, whereas another feeds into a G_i_-mediated pathway (Wetzker and Bohmer, 2003). Although by far the majority of evidence suggests a preference for interacting with G_q_, some previous work has shown V_1A_ receptors coupling to G_iα3_ in quiescent fibroblasts and in hepatocytes (Strakova *et al*, 1997; Abel *et al*, 2000) There is also evidence that GRP receptors couple to G_i_ in pancreatic acinar cells (Profrock *et al*, 1992), and that G_i_ mediates bombesin-stimulated chemotaxis in monocytes (Djanani *et al*, 2003). Our data suggest that in the presence of substance-P analogues, the neuropeptide receptors exist in a conformation that is unable to activate G_q_ but which is able to interact with G_i_ and activate ERK. These analogues may act at a common region of the GPCRs perhaps at the receptor/G-protein interface, which favours coupling to G_i_. Although activation of ERK is normally associated with a mitogenic response as is seen with the neuropeptides, deregulated ERK activation has also been shown to cause disruption of the cell cycle, growth arrest and apoptosis. In SCLC-activated raf-1, the upstream regulator of ERK causes growth arrest (Ravi *et al*, 1998). It is our hypothesis that deregulated ERK coupled with a decrease in intracellular Ca^2+^ are important factors in the growth inhibitory/apoptosis inducing activity of substance-P analogues.

In summary, the results presented here show that neuropeptide receptor expression leads to the development of a transformed phenotype in CHO epithelial cells. These cells display increased sensitivity to substance-P analogues that parallels the situation observed in SCLC cells, which develop chemoresistance *in vivo*. These analogues activate GRP and V_1A_ receptors in such a way as to cause activation of only a subset of possible downstream signals. They are competitive antagonists for PI hydrolysis mediated via G_q_ but have agonist activity that results in activation of ERK via G_i_. This presents a novel pharmacological mechanism whereby substance-P analogues utilise the normally mitogenic neuropeptide receptors to transduce an antiproliferative and apoptogenic cell signal. Moreover, the concentration of SPG used in this study was detected in patient plasma following administration with no noticeable toxicity (Clive *et al*, 2001), suggesting therapeutic doses can be achieved clinically. Development of these agents may provide a unique opportunity to treat SCLC and may be of particular benefit for tumours that have developed resistance to chemotherapy.
